# Combined Simulated Annealing Algorithm for the Discrete Facility Location Problem

**DOI:** 10.1100/2012/576392

**Published:** 2012-09-19

**Authors:** Jin Qin, Ling-lin Ni, Feng Shi

**Affiliations:** ^1^School of Traffic and Transportation Engineering, Central South University, Changsha 410075, China; ^2^Business Administration College, Zhejiang University of Finance & Economics, Hangzhou 310018, China

## Abstract

The combined simulated annealing (CSA) algorithm was developed for the discrete facility location problem (DFLP) in the paper. The method is a two-layer algorithm, in which the external subalgorithm optimizes the decision of the facility location decision while the internal subalgorithm optimizes the decision of the allocation of customer's demand under the determined location decision. The performance of the CSA is tested by 30 instances with different sizes. The computational results show that CSA works much better than the previous algorithm on DFLP and offers a new reasonable alternative solution method to it.

## 1. Introduction

The classical facility location problem (FLP) is one of the most important models in combinatorial optimization, which is to determine the number and locations of the facilities and allocate customers to these facilities in such a way that the total cost is minimized. The FLP may be the most critical and most difficult decision in the designing of an efficient supply chain for the facilities are costly and difficult to reverse after being located. The problem is also encountered in other areas such as material distribution, transportation network, and telecommunication network. 

The FLP can be classified in two categories as discrete problem and continuous problem according to whether the sets of demand points and facility locations are finite. The discrete facility location problem (DFLP) assumes that the solution space is discrete and generally the facilities are located on the nodes of the network, which brings a lot of complexities to the problem. And many practical problems without facilities to locate, such as cluster analysis, machine scheduling, economic lot sizing, portfolio management, and computer network design, can also be modeled as DFLP [1]. Due to its strategic nature, DFLP has been widely studied by researchers over many years, especially developing solution methods for the DFLP has been a hot topic of research for the last 30 years. Many successful contributions of the DFLP have been reported both in theory and in practice. The Daskin [2] and Melo et al. [1] provided thorough reviews of the DFLP. 

The DFLP is a NP-hard problem, and this nature makes the exact algorithms only for small problems and heuristics the natural choice for larger instances. Therefore much attention has been focused on designing heuristics algorithms with good performance. Following the first heuristics algorithm presented by Shmoys et al. [3], there was a long list of work on designing heuristics algorithms for this problem over the years. As a result, four basic algorithms with different features have been emerged, namely, LP-rounding [3–8], primal-dual [9–11], dual-fitting [12–14], and local search [15–18]. Although LP-rounding could be used to design algorithm with better results than other three, it is noncombinatorial in nature and needs more CPU time. Primal-dual and dual-fitting method could be adapted to solve variants of the FLP but are less robust. Recently some applications have proved that local search is more powerful on the hard DFLP [16]. And along with the development of the computation method, more and more researchers use the heuristic algorithms based on the local search to solve the problem, such as simulated annealing [19–21] and genetic algorithm [20, 22].

In this paper, a general algorithmic framework of combined simulated annealing (CSA) is developed for the DFLP, and its performance is compared with other existed solution methods.

The rest of this paper is organized as follows. In Section 2, we describe the general model of DFLP. The basic procedures of the SA and CSA are presented in Sections 3 and 4 respectively.  Section 5 shows the computational results on test instances, and Section 6 concludes the paper.

## 2. The Formulation of DFLP Model

We start by giving the mathematical formulation of a general model for DFLP with a minimized objective function. In the model, *I* = {1,2,…, *n*} is used to represent the set of the customers and *J* = {1,2,…, *m*} is used to represent the set of the potential location sites. *f*_*j*_ is used to represent the fixed cost of opening a facility at site *j* ∈ *J*, *v*_*j*_ is the service capacity of facility at site *j*, *d*_*i*_ is the demand of customer *i* ∈ *I*, and *c*_*ij*_ is the cost of serving one unit of demand at customer *i* from site *j*, in other word, the unit variable shipping cost between customer *i* from site *j*. We reasonably assume that *c*_*ij*_ ≥ 0, *f*_*j*_ ≥ 0, and *v*_*j*_ ≥ 0 for all *i* ∈ *I*, for all *j* ∈ *J*. 

And two binary variables are set as:

(1) $$\begin{array}{c} X_{j}=\{\begin{array}{cc} 1 & \text{if}\;\text{a}\;\text{facility}\;\text{setup}\;\mathrm{on}\;\text{site}\;\;i;\\ 0 & \text{otherwise}; \end{array}\\ Y_{ij}=\{\begin{array}{cc} 1 & \text{if}\;\text{facility}\;j\;\text{serves}\;\text{customer}\;i;\\ 0 & \text{otherwise}. \end{array} \end{array}$$

Then the general model of DFLP could be stated as the following linear mixed-integer program:

(2) min⁡ Φ=∑j=1mfjXj+∑i=1n∑j=1mdicijYij

(3) Subject  to ∑j=1mYij=1 ∀i∈I

(4) ∑i=1ndiYij≤vjXj ∀j∈J

(5) Yij−Xj≤0 ∀i∈I,  ∀j∈J

(6) Xj∈{0,1} ∀j∈J

(7) Yij∈{0,1} ∀i∈I,  ∀j∈J.

The objective function (2) is to minimize the total system cost, including the location cost and the shipment cost. Constraint (3) is the demand constraint, which makes the demand of each customer be met; (4) is the variable upper bound constraint; (5) is the capacity constraint of facility; (6) and (7) are standard binary integrality constraints.

## 3. Simulated Annealing Algorithm for DFLP

Kirkpatrick et al. [23] introduced the concept of simulated annealing (SA) algorithmin 1983, which is a stochastic optimization technique. To be specific, SA is a probabilistic heuristic for the global optimization problems of finding a good approximation to the global optimum of a given objective function in the search space. It is often used when the solution space is discrete. In the searching process, the SA accepts not only better but also worse neighboring solutions with a certain probability. This means that the SA has the ability to escape from the local minima. Therefore, it can find high-quality solutions that do not strongly depend upon the choice of the initial solution compared to other local search algorithms. And its another advantage over the other heuristic algorithms is the ease of implementation. So we adopted SA as the basic solution method to solve the DFLP. 

In last 30 years, SA has been studied widely and used extensively in many optimization problems [24–29], which have proved that SA is an effective tool for approximating globally optimal solutions to many NP-hard optimization problems.

In order to describe the procedure of the SA, **S**, **S**′, **S**′′, and $\overset{-}{S}$ are used to represent the different feasible solutions of the model; *D*(*T*_*i*_) is the cooling function of temperature, in which *T*_*i*_ is the current temperature value. *T*_*f*_ is the stop temperature value. *N* is the maximum iteration number at each temperature value. Φ(**S**) is used to represent the objective function value of the solution **S**. According to Jayaraman and Ross [19], the SA for DFLP could be given as follows.

**Step 1 (initialization)**

Set iteration counter *i* = 1. Generate an initial feasible solution **S** and regard **S** as the optimal solution. Set the initial temperature *T*_*i*_ and the stop temperature *T*_*f*_ are specified. Define the cooling function *D*(*T*_*i*_).

**Step 2 (generate a feasible neighboring solution)**

Perform the neighboring function on current solution **S** and get the new neighboring solution **S**′.

**Step 3 (evaluate current solution with neighboring solution)**

If the objective function value of the new solution **S**′ is no less than that of the current solution **S**, namely, Φ(**S**′) ≥ Φ(**S**), then proceed to Step 4; otherwise, if Φ(**S**′) < Φ(**S**), then **S** = **S**′, proceed to Step 5.

**Step 4 (examine metropolis condition)**

Determine the difference Δ*C* between the incumbent solution **S** and the neighboring solution **S**′, as ΔΦ = Φ(**S**′) − Φ(**S**). Generate a random number *ρ* from the interval (0,1), if *ρ* < exp⁡(−ΔΦ/*T*_*i*_), then **S** = **S**′. Proceed to Step 5.

**Step 5 (check increment counters)**

Set *i* ← *i* + 1. If *i* ≤ *N*, then return to Step 2. Otherwise proceed to Step 6.

**Step 6 (adjust temperature)**

Adjust temperature by the cooling function, Mathematically this is *T*_*i*_ ← *D*(*T*_*i*_).

**Step 7 (convergence check)**

If *T*_*i*_ ≥ *T*_*f*_, then reset *i* = 1 and return to Step 2. Otherwise, stop and output the optimal solution **S**.

## 4. Combined Simulated Annealing (CSA)

The solution of DFLP includes two parts: *X*_*j*_s and *Y*_*ij*_s. *X*_*j*_ denotes whether or not open the facility at site *j*, while *Y*_*ij*_ denotes the service demand allocation. The two variables are interdependent and interactional. As each demand must allocate to an opening facility, we could can conclude that the variable *Y*_*ij*_ is subject to the variable *X*_*j*_. This relation also can be seen from the constrain (4) in the model in Section 1.

CSA works in two layers as internal layer and external layer to solve the problem. The internal layer subalgorithm (ILSA) would optimize the facility location decision variable *X*_*j*_. The external layer subalgorithm (ELSA) would perform the allocation optimization under fixed *X*_*j*_s which determined in the internal layer. In each layer the SA is used and they make up of the CSA. The method that divides the problem into two layers could make the search in the procedure explores in smaller solution space each time, so it increases the probability of obtaining the global optimal solution.

Some parameters should be initialized before the performance of CSA, including the initial temperature and stop temperature, cooling ruler of the temperature, iteration maximum in each temperature. The initialization of the parameters could be determined in similar ways to these in the SA. And the **S**, **S**′, **S**′′, $\overset{-}{S}$ are also used to represent the different feasible solutions here.

In addition, CSA must start with an initial solution or with a solution produced using a heuristic. In this work, we use the randomly generated initial solution, which proposed in Qin et al. [30].

### 4.1. Neighboring Functions

Similar to the SA, the CSA algorithm is an iterative search procedure based on the neighboring function. The quality of the optimal solution is very sensitive to the way that the candidate solutions are selected. Thus, the neighboring function is crucial to the good performance of the CSA algorithm. 

The ELSA would optimize the facility location decision. So the neighboring function of the ELSA to modify the configuration of the current solution and generate a neighboring solution could perform three different operations:

1. If the number of the located facilities is less than the allowed maximum *M* (∑_*j*=1_^*m*^ *X*_*j*_ < *M*), then select a candidate site *i* which satisfies *X*_*j*_ = 0 in current solution *S* randomly and set *X*_*j*_ = 1, namely, there would locate a new facility.
2. If the number of the locate facility no less than 1 (∑_*j*=1_^*m*^ *X*_*j*_ ≥ 1), then select a site *j* randomly which satisfies *X*_*j*_ = 1 in solution *S* and set *X*_*j*_ = 0, namely, there would close a open facility.
3. Select a site *j* which satisfies *X*_*j*_ = 0 and another site *j*′ which satisfies *X*_*j*′_ = 1 in current solution *S*, then set *X*_*j*_ = 1 and *X*_*j*′_ = 0.

In the implementation of the ELSA, we could select only one operation from the above three operations to perform the neighboring function each time. And after implementing the operation, it should allocate the customers' demand to the opened facilities again, as generate an initial solution again.

ILSA is to determine the demand allocation decision. According to the features of the allocation decision, there are two operations to generate the neighboring solutions in the ILSA.

1. Select two allocation variables *Y*_*ij*_ and *Y*_*ij*′_ that satisfy *Y*_*ij*_ = 1 and *Y*_*ij*′_ = 0; then set *Y*_*ij*′_ = 1, *Y*_*ij*_ = 0, namely, it allocates the demand of customer *i* from facility *j* to facility *j*′.
2. Exchange the facilities which serve two customers re spectively with each other. To be specific, select four allocation variables as *y*_*i*_1_*j*_1__ = 1, *y*_*i*_2_*j*_2__ = 1, *y*_*i*_1_*j*_2__ = 0, *y*_*i*_2_*j*_1__ = 0, then set *y*_*i*_1_*j*_2__ = 1, *y*_*i*_2_*j*_1__ = 1, *y*_*i*_1_*j*_1__ = 0, *y*_*i*_2_*j*_2__ = 0.

Similarly, the neighboring function of the ILSA could select only one operation to perform each time. In addition, it must ensure that demands of all customers must be satisfied and the facilities have no capacity violations exist. Otherwise, it should return to the old solution and reselect an operation to perform.

### 4.2. The Procedure of CSA

The following is a step-by-step description of the procedure of CSA.

**Step 1 1 (initialization)**

Set iteration counter *i* = 1, *k* = 1. Set the initial temperature *T*_*i*_, and stop temperature *T*_*f*_, the initial feasible solution is **S**, and let **S** be the optimal solution at the same time. Define the cooling function *D*(*T*_*i*_). Generate a feasible solution by randomly allocation with capacity restricted.

**Step 2 (check feasibilities)**

The method now checks the demand allocations to ensure that no capacity violations exist. The demands of the customers are also checked. If the solution **S** is not feasible, we should return to Step 1.

**Step 3 (generate a neighboring solution)**

Perform the external layer neighboring function on solution **S**, and generate a neighboring feasible solution **S**′.

**Step 4 (perform ILSA)**

*Step *4.1. Set *k* = 1. Regard solution **S**′ as the initial solution and the current optimal solution.

*Step *4.2. Perform the internal layer neighboring function on solution **S**′, and generate a neighboring feasible solution **S**′′.

*Step *4.3. If Φ(**S**′′) < Φ(**S**′), then set **S**′ = **S**′′; otherwise, generate a random number *ρ* from (0,1), if *ρ* < exp( − (Φ(**S**′′) < Φ(**S**′))/*T*_*i*_), then set **S**′ = **S**′′.

*Step *4.4. Consider *k* ← *k* + 1. If *k* ≤ *N*_2_, then return to Step 2. Otherwise proceed to Step 5.

*Step *4.5. Stop and output the optimal solution **S**′, return to the ELSA.

**Step 12 (save the global optimal solution)**

If $\Phi \left(S^{\prime }\right)<\Phi \left(\overset{-}{S}\right)$, then $\overset{-}{S}=S^{\prime }$.

**Step 6 (evaluate current solution)**

Evaluate current solution with neighboring solution. If Φ(**S**′) < Φ(**S**), then let **S** = **S**′ proceed to Step 7; otherwise, proceed to Step 7.

**Step 7 (examine metropolis condition)**

A random number *ρ* is generated from (0,1), if *ρ* < exp⁡(−(Φ(**S**′) − Φ(**S**))/*T*_*i*_) then **S** = **S**′. Proceed to Step 8.

**Step 8 (increment counters)**

Set *i* ← *i* + 1. If *i* ≤ *N*_1_, then return to Step 3; otherwise, proceed to Step 9.

**Step 9 (adjust temperature)**

Adjust temperature by the cooling function: *T*_*i*_ ← *D*(*T*_*i*_).

**Step 10 (convergence check)**

If *T*_*i*_ ≥ *T*_*f*_, then reset *i* = 1 and return to Step 3. Otherwise, stop and output the optimal solution $\overset{-}{S}$.

*N*_1_, *N*_2_ are the given maximum iteration number in ELSA and ILSA respectively. The Step 5 is to save the global optimal solution that has been found so far in the CSA. This operation does not take the acceptance probability of worse solution into consideration. So it could help the algorithm avoid losing the global optimal solution.

## 5. Computational Experiments

To assess the practical effectiveness of the proposed CSA algorithm, we use 30 instances with different sizes as benchmark problems. Twelve “Capacitated warehouse location” instances were given by Beasley [31], which are publicly available from the OR-Library and could be downloaded directly from the website: http://people.brunel.ac.uk/~mastjjb/jeb/orlib/capinfo.html. The other 18 instances were proposed by Ghosh [32].

To perform the CSA, the initial temperature *T*_1_ could be set equal to the objective function value of the initial solution. The cooling ruler is equal-ratio cooling, and the cooling ratio *α* = 0.95. The iteration maximum *N*_1_ = *m*, *N*_2_ = 5*n*, the stop temperature value *t*_*f*_ = 0.001. The SA is used to solve the instances too, and its iteration maximum under the same temperature is 5*mn*; other parameters are same as CSA. So the total iteration numbers in the SA and CSA are equal. We also use the randomly generated method to find an initial feasible solution.

The model and CSA were implemented in Visual C# 2010. A personal computer with Intel E5800 CPU, 2G RAM and Windows XP Profession operating system was used for all tests.

**Example 1 (OR-Library Instances)**

The instances in OR-Library are more than 150, and we selected 12 instances with different size to be solved by the CSA and compared with theirs optimal solution (all instances have been exactly solved by the Lindo software and provide their optimal solutions by the author).

Computational results of these OR-Library instances are reported in Table 1. Each row of the table gives the results of one individual instance. The instance names are originally used in the OR-Library. The gap in the table represented the relative error between the result and the optimal solution.

It can be observed from Table 1 that the CSA found optimal solutions for all these instances without any exception, while the SA didn't find anyone. The CPU time used by SA for each instance is from 3 to 8 times of that used by CSA.

**Figure 1:**
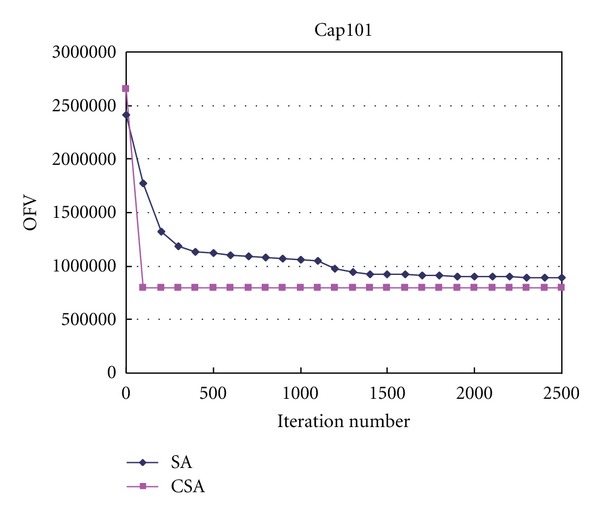
OFV versus iteration number in Cap101.

**Figure 2:**
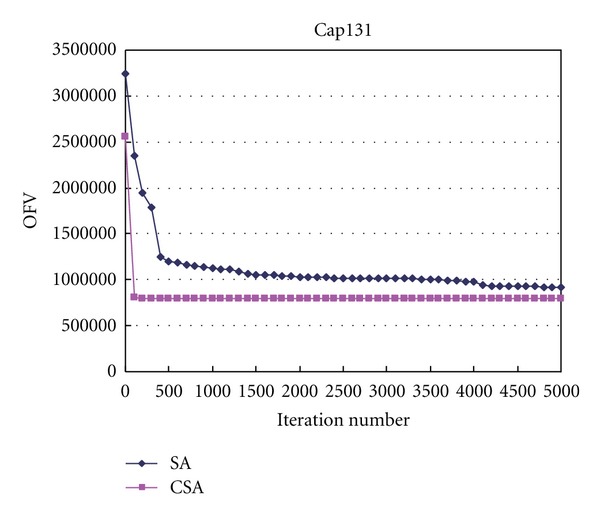
OFV versus iteration number in Cap131.

The computation process of instances Cap101 and Cap131 with CSA and SA are depicted in Figures 1 and 2. The OFV is the objective function value. As shown in the figures, we can find that the convergence speed of CSA is more quickly than that of SA. The iteration time for converging to the optimal solution used by SA for each instance is from 10 to 25 times of that used by CSA.

**Example 2 (The Ghosh Instances)**

The Ghosh instances are 90 instances in total, and with *n* = *m* on all cases. These instances of the same size are divided into two categories, symmetric, where the transportation cost *c*_*ij*_ = *c*_*ji*_ holds, and asymmetric, where *c*_*ij*_ = *c*_*ji*_ does not necessarily hold. Each group contains three values of *m* × *n*: 250 × 250, 500 × 500, and 750 × 750. The instances in each category are further divided into three instance classes, and they differ in the range of values from which opening costs *f*_*j*_ are drawn, which could be chosen from the [100,200] (Range A), [1000,2000] (Range B), and [10000,20000] (Range C) randomly.

The optimal solutions of the Ghosh instances were not presented, but there used to be several methods to solve the instances and compared the results with each other in the literature [33]. And we compare the results of the CSA with them as reported in Table 2, in which we reported the results of the instances obtained by hybrid, CLM, GTS, TS, and CSA.

As shown in Table 2, for all instances, CSA found solutions better than those of other methods with only five exceptions, and found solutions with the same result for symmetric instance with *m* × *n* = 500 × 500 in type C. Even in the five exceptions, the gaps between the solutions were found by CSA and the best solutions found by other methods are very small, and the maximal relative gap is only 3.1% in asymmetric instance with *m* × *n* = 250 × 250 in type C.

## 6. Conclusions

The DFLP is to determinate the facility location and demand allocation so as to minimize the total cost, based on the demands of customers satisfied without violating the capacity restriction of any facility. The CSA was proposed for the DFLP, which is a two-layer algorithm. Its external layer subalgorithm optimizes the facility location decision, while the internal layer subalgorithm optimizes the demand allocation under the fixed facility location which is determined by the external layer. The each local search process in CSA focuses on the smaller solution space, which could not only increase the probability of obtaining the global optimal solution, but also save computational time.

The performance of CSA is evaluated with 30 instances with different sizes. Those instances are solved by CSA in a very reasonable amount of time, and the solutions are compared with that of previous studies in the literature. It is showed that the new algorithm could give better results (or at least same) than the others for nearly all instances. Hence, the CSA works much better than the previous work and offers a reasonable alternative solution method to the DFLP.

## Figures and Tables

**Table 1:** Comparison for OR-Library instances.

Instances			SA			CSA		
Name	Size (*m* × *n*)	Optimal solution	Result	Gap (%)	CPU time^(S)^	Result	Gap (%)	CPU time^(S)^
Cap71	16 × 50	932615.750	1192413.575	6.995	4.233	932615.750	0	0.921
Cap72	16 × 50	977799.400	1012970.190	5.970	4.690	977799.400	0	1.599
Cap73	16 × 50	1010641.450	1100470.190	8.179	4.435	1010641.450	0	0.732
Cap74	16 × 50	1034976.975	1212970.191	12.276	6.438	1034976.975	0	1.499
Cap101	25 × 50	796648.440	886494.475	9.250	12.552	796648.440	0	2.192
Cap102	25 × 50	854704.200	998953.625	15.698	13.652	854704.200	0	2.612
Cap103	25 × 50	893782.1125	1002291.150	11.412	12.002	893782.1125	0	1.798
Cap104	25 × 50	928941.750	1177291.150	24.333	18.263	928941.750	0	5.048
Cap131	50 × 50	793439.5620	856571.455	6.607	54.688	793439.562	0	24.723
Cap132	50 × 50	851495.3250	1011571.450	16.732	45.563	851495.325	0	19.803
Cap133	50 × 50	893076.7120	1219071.450	32.485	56.549	893076.712	0	8.842
Cap134	50 × 50	928941.7500	1374071.452	41.690	49.156	928941.750	0	14.972

**Table 2:** Comparison for the Ghosh instances.

Size (*m* × *n*)	Instances		Hybrid		CLM		GTS		TS		CSA	
	Class	Range	Result	CPU time^1^	Result	CPU time^1^	Result	CPU time^1^	Result	CPU time^1^	Result	CPU time
250 × 250	Symmetry	A	257806.8	4.328	257895.2	86.482	257832.6	2.828	257805.0	3.687	**257293.0**	66.652
250 × 250	Symmetry	B	276035.2	7.774	276352.2	34.634	276185.2	5.628	276035.2	5.347	**275560.0**	69.339
250 × 250	Symmetry	C	***333671.6***	8.702	***333671.6***	69.458	333820.0	9.878	***333671.6***	10.384	334127.0	70.588
250 × 250	Asymmetry	A	257923.4	4.636	258032.6	86.506	257978.4	2.618	257917.8	3.487	**257179.0**	81.706
250 × 250	Asymmetry	B	276053.2	8.082	276184.2	33.688	276467.2	5.790	276053.2	5.501	**275111.0**	75.473
250 × 250	Asymmetry	C	332897.2	7.776	333058.4	89.990	333237.6	9.196	**332897.2**	9.736	343270.0	70.329
500 × 500	Symmetry	A	511196.4	27.644	511487.2	946.028	511383.6	15.616	511180.4	14.835	**511113.0**	541.772
500 × 500	Symmetry	B	***537912.0***	34.196	538685.8	294.656	538480.4	31.432	***537912.0***	29.860	***537912.0***	579.244
500 × 500	Symmetry	C	621059.2	40.376	621172.8	437.462	621107.2	71.106	621059.2	67.551	**620112.0**	663.167
500 × 500	Asymmetry	A	511145.0	20.232	511393.4	921.208	511251.6	13.760	511140.0	13.072	**511097.0**	543.235
500 × 500	Asymmetry	B	537863.4	31.300	538421.0	311.344	538144.0	34.748	537847.6	33.011	**535665.0**	358.807
500 × 500	Asymmetry	C	621463.8	47.790	621990.8	388.210	621811.8	72.064	**621463.8**	68.461	630861.0	509.046
750 × 750	Symmetry	A	763706.6	49.214	763978.0	3650.662	763830.8	39.812	763693.4	37.821	**763417.0**	1250.775
750 × 750	Symmetry	B	796632.2	92.886	797173.4	1583.170	796919.0	93.352	796571.8	88.684	**794970.0**	1517.086
750 × 750	Symmetry	C	900272.0	113.640	900785.2	1194.534	901158.4	229.914	**900158.6**	218.418	926358.0	1387.104
750 × 750	Asymmetry	A	763731.2	59.130	764019.2	3658.588	763836.6	39.650	763717.0	34.667	**763498.0**	1548.608
750 × 750	Asymmetry	B	796396.8	73.322	796754.2	1606.778	796859.0	95.430	796374.4	90.660	**793668.0**	1598.631
750 × 750	Asymmetry	C	**900193.2**	112.994	900349.8	1325.812	900514.2	236.902	**900193.2**	205.060	919453.0	1337.671

Note: CPU time^1^ is on Sun Enterprise 3000 Server (4 × CPU, 6 G memory).
